# The differential impact of a 6-*versus* 12-month pharmacist-led interprofessional medication adherence program on medication adherence in patients with diabetic kidney disease: the randomized PANDIA-IRIS study

**DOI:** 10.3389/fphar.2024.1294436

**Published:** 2024-01-24

**Authors:** Carole Bandiera, Jennifer Dotta-Celio, Isabella Locatelli, Dina Nobre, Grégoire Wuerzner, Menno Pruijm, Faiza Lamine, Michel Burnier, Anne Zanchi, Marie Paule Schneider

**Affiliations:** ^1^ School of Pharmaceutical Sciences, University of Geneva, Geneva, Switzerland; ^2^ Institute of Pharmaceutical Sciences of Western Switzerland, University of Geneva, Geneva, Switzerland; ^3^ Center for Primary Care and Public Health (Unisanté), University of Lausanne, Lausanne, Switzerland; ^4^ Service of Nephrology and Hypertension, Department of Medicine, Lausanne University Hospital and University of Lausanne, Lausanne, Switzerland; ^5^ Service of Endocrinology, Diabetes and Metabolism, Department of Medicine, Lausanne University Hospital, Lausanne, Switzerland

**Keywords:** medication adherence, electronic adherence monitoring, adherence interventions, diabetes complication, diabetic kidney disease, nephropathy, interprofessionality, digital technology

## Abstract

**Background:** For every 100 patients with diabetes, 40 will develop diabetic kidney disease (DKD) over time. This diabetes complication may be partly due to poor adherence to their prescribed medications. In this study, we aimed to evaluate the differential impact of a 6- *versus* 12-month pharmacist-led interprofessional medication adherence program (IMAP) on the components of adherence (i.e., implementation and discontinuation) in patients with DKD, during and after the intervention.

**Methods:** All included patients benefited from the IMAP, which consists in face-to-face regular motivational interviews between the patient and the pharmacist based on the adherence feedback from electronic monitors (EMs), in which the prescribed treatments were delivered. Adherence reports were available to prescribers during the intervention period. Patients were randomized 1:1 into two parallel arms: a 12-month IMAP intervention in group A *versus* a 6-month intervention in group B. Adherence was monitored continuously for 24 months post-inclusion during the consecutive intervention and follow-up phases. In the follow-up phase post-intervention, EM data were blinded. Blood pressure was measured by the pharmacist at each visit. The repeated measures of daily patient medication intake outcomes (1/0) to antidiabetics, antihypertensive drugs, and statins were modeled longitudinally using the generalized estimated equation in both groups and in both the intervention and the follow-up phases.

**Results:** EM data of 72 patients were analyzed (34 in group A and 38 in group B). Patient implementation to antidiabetics and antihypertensive drugs increased during the IMAP intervention phase and decreased progressively during the follow-up period. At 12 months, implementation to antidiabetics was statistically higher in group A *versus* group B (93.8% *versus* 86.8%; Δ 7.0%, 95% CI: 5.7%; 8.3%); implementation to antihypertensive drugs was also higher in group A *versus* B (97.9% *versus* 92.1%; Δ 5.8%, 95% CI: 4.8%; 6.7%). At 24 months, implementation to antidiabetics and antihypertensive drugs remained higher in group A *versus* B (for antidiabetics: 88.6% *versus* 85.6%; Δ 3.0%, 95% CI: 1.7%; 4.4% and for antihypertensive drugs: 94.4% *versus* 85.9%; Δ 8.5%, 95% CI: 6.6%; 10.7%). No difference in pharmacy-based blood pressure was observed between groups. Implementation to statins was comparable at each time point between groups. Three patients discontinued at least one treatment; they were all in group B. In total, 46% (16/35) of patients in the 12-month intervention *versus* 37% (14/38) of patients in the 6-month intervention left the study during the intervention phase, mainly due to personal reasons.

**Conclusion:** The IMAP improves adherence to chronic medications in patients with DKD. The longer the patients benefit from the intervention, the more the implementation increases over time, and the more the effect lasts after the end of the intervention. These data suggest that a 12-month rather than a 6-month program should be provided as a standard of care to support medication adherence in this population. The impact on clinical outcomes needs to be demonstrated.

**Clinical Trial Registration:** Clinicaltrials.gov, identifier NCT04190251_PANDIA IRIS.

## 1 Introduction

### 1.1 Background

The pandemic of diabetic disease keeps growing worldwide. It is estimated that in 2021, 537 million adults were living with diabetes and 6.7 million died from this disease. It is expected that 783 million patients will be diagnosed with diabetes by 2045 (International-Diabetes-Federation, 2021). The global health economic burden of adult patients with diabetes keeps rising, reaching USD 966 billion worldwide in 2021 (International-Diabetes-Federation, 2021). Thus, diabetes is an urgent public health concern and an important economic burden for the healthcare systems. Several types of diabetes exist, all characterized by hyperglycemia. Uncontrolled hyperglycemia severely degrades tissues and organs, leading to microvascular (i.e., retinopathy, kidney disease, and neuropathy) and macrovascular complications (i.e., atherosclerosis) (Fowler, 2008). Among these complications, diabetic kidney disease (DKD) is characterized by a chronically reduced estimated glomerular filtration rate (eGFR) below 60 mL/min/1.73 m^2^ (in 70% of patients) (Sheen and Sheu, 2014) and/or the presence of increased albuminuria (Gheith et al., 2016). DKD is the leading cause of end-stage kidney disease (Stewart et al., 2004; Johansen et al., 2022) defined as an eGFR of less than 15 mL/min/1.72 m^2^ (MFMER, 2023). It is estimated that 40% of patients with diabetes will develop DKD over time (Gross et al., 2005; Gheith et al., 2016).

The goals of pharmacological treatments for diabetes focus on delaying the progression of the renal impairment and preventing cardio-renal events and complications by intensively controlling blood pressure, lipids, and glycemic blood levels and providing cardio-protection with evidence-based therapies. As a consequence, patients with DKD are polypharmacy patients, which may contribute to treatment nonadherence.

Medication adherence is described by three interrelated and quantifiable phases, following ideally a shared decision-making process regarding prescribing: initiation (i.e., first dose taken), implementation (i.e., the extent to which the patient takes the treatment as prescribed), and discontinuation (i.e., the patient stops taking the treatment earlier than planned by the prescriber) (Vrijens et al., 2012). Treatment persistence is the time between initiation and discontinuation (Vrijens et al., 2012). Literature reports that 40% of patients with DKD are not adherent to their medications (Williams et al., 2012; Kefale et al., 2018; Balasubramaniam et al., 2019), while medication nonadherence leads to poor clinical outcomes and increases mortality (Chang et al., 2015; Shani et al., 2017; Paranjpe et al., 2022). Medication adherence must become a priority for interprofessional healthcare teams. However, studies evaluating interventions aiming to improve adherence in patients with DKD are scarce, and their impact on adherence and clinical outcomes remains limited (Williams et al., 2012; Helou et al., 2016; Zimbudzi et al., 2018). As a consequence, the type and duration of interventions to improve adherence are largely unknown in this patient population.

The PANDIA-IRIS (*Patients diabétiques et insuffisants rénaux: un programme interdisciplinaire de soutien à l’adhésion thérapeutique*) study was developed at the community pharmacy of the Center for Primary Care and Public Health *Unisanté* to support medication adherence in patients with DKD. The intervention consists in a pharmacist-led interprofessional medication adherence program (IMAP), implemented since 1995 at the community pharmacy of *Unisanté*, aiming to support medication adherence in chronically ill patients (Lelubre et al., 2015).

### 1.2 Objectives

The main objective of the PANDIA-IRIS study was to evaluate the differential impact of a 6-month *versus* 12-month pharmacist-led IMAP on implementation and persistence to antihypertensive drugs, antidiabetics, statins, and aspirin in patients with DKD at different time points, i.e., at 6, 12, 18, and 24 months post-inclusion. The secondary objective was to evaluate the impact of the intervention on the United Kingdom Prospective Diabetes Study (UKPDS) and the Action in Diabetes and Vascular disease: Preterax and Diamicron Modified-Release Controlled Evaluation (ADVANCE) clinical scores.

### 1.3 Outcomes

The medication intake is a binary variable (1 = correct intake; 0 = incorrect intake) measured using an electronic monitor (EM) in a patient at each day of the monitoring period. To be considered optimal, the medication intake has to be correct (=1) for every EM used. On each day, medication implementation is the proportion of patients with a correct medication intake among patients still under observation on that day. Persistence to treatment is characterized by the time between study initiation and treatment discontinuation (= 1) for each patient. The secondary outcomes were the ADVANCE and UKDPS clinical scores, systolic and diastolic blood pressures measured at each visit at the pharmacy, and the number of patients with an electronic medication implementation of less than 30% for at least one medication throughout two successive pharmacy visits during the post-intervention phase.

### 1.4 Hypothesis and research questions

We hypothesized that patients in both groups would benefit from the IMAP, yet the impact of the intervention on medication adherence, i.e., implementation and persistence, would be higher and would last longer post-intervention in participants included in the IMAP for 12 months (group A) compared to patients who benefited from the IMAP during 6 months (group B). We hypothesized that during the follow-up period post-intervention, patients included in group A would maintain a higher implementation compared to patients included in group B.

## 2 Methods

### 2.1 Ethical considerations and guidelines

The PANDIA-IRIS study was approved by the local Ethics Committee “*Commission cantonale d'éthique de la recherche sur l'être humain*” (Vaud, Switzerland, ID 2016-01674). All patients signed an informed consent form to participate in this study. The study was conducted in accordance with the Declaration of Helsinki. Both the ESPACOMP Medication Adherence Reporting Guidelines (EMERGE) (De Geest et al., 2018) and Consolidated Standards of Reporting Trials (CONSORT) guidelines (Schulz et al., 2010) were used to report findings.

### 2.2 Design of the PANDIA-IRIS medication adherence study

The protocol of the PANDIA-IRIS study has been published elsewhere (Bandiera et al., 2021). The PANDIA-IRIS study was monocentric, open, and randomized. Patients were recruited from April 2016 to October 2020 from the Service of Nephrology and Hypertension, the Service of Endocrinology, Diabetes and Metabolism of the Lausanne University Hospital (*Centre hospitalier universitaire vaudois*, CHUV), and at the policlinic of the Center for Primary Care and Public Health *Unisanté*, located in the same hospital complex. The first patient was included in April 2016, and the data collection ended on the last visit of the last patient in December 2022.

Eligible patients were adults with a diagnosis of diabetes—either type 2, type 1, latent autoimmune diabetes in adults (LADA) or glucocorticoid-induced—with chronic kidney disease (an eGFR of less than 60 mL/min/1.73 m^2^). In October 2019, an amendment was accepted by the local Ethics Committee to expand recruitment from adults diagnosed with type 2 diabetes only to adults with the four types of diabetes listed above in order to increase recruitment.

**FIGURE 1:**
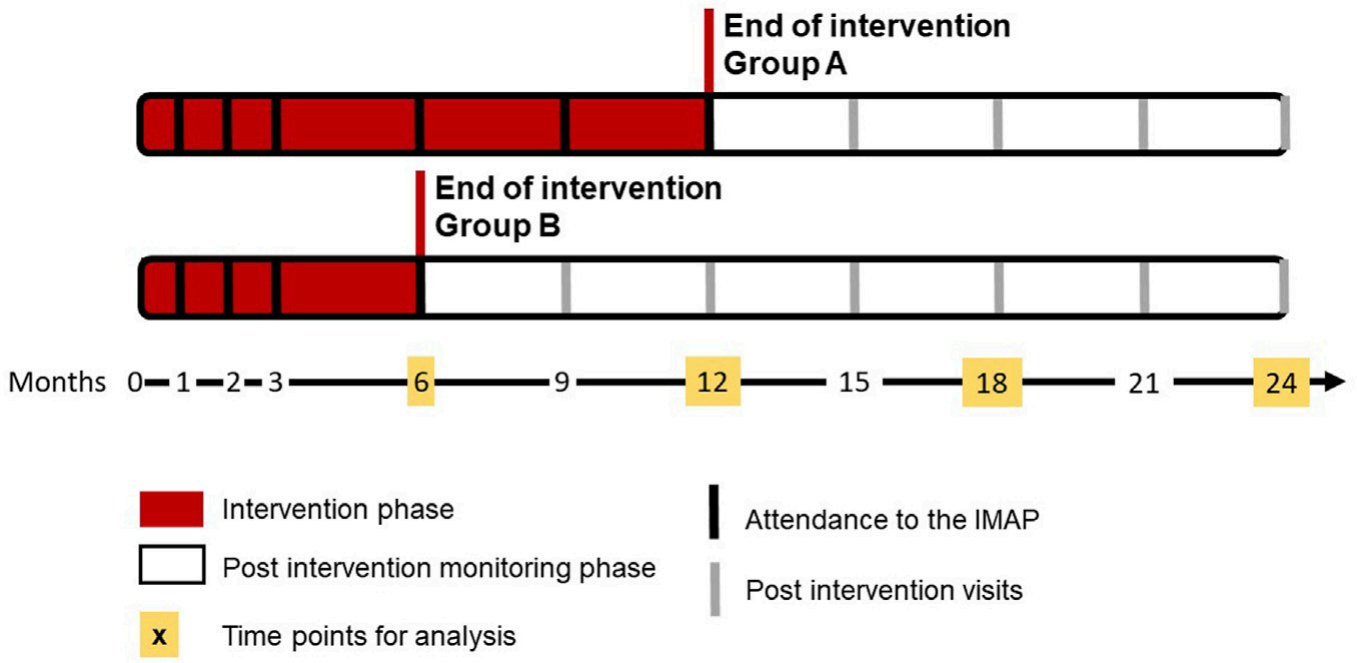
Design of the PANDIA-IRIS study, adapted from Bandiera et al., 2021. IMAP, interprofessional medication adherence program.

Patients were excluded if they did not self-manage their treatments (i.e., home care services and nursing homes) or had cognitive disorders. Patients who were pregnant or had an active cancer were also excluded. The calculation of the sample size was detailed in the published protocol (Bandiera et al., 2021) and showed that 72 patients (36 patients in each group) should be included. Enrolled patients were randomized 1:1 at inclusion into two parallel arms, each lasting 24 months. Participants in the first arm received the intervention for 12 months (group A) *versus* 6 months (group B) in the second arm (Figure 1 adapted from Bandiera et al. (Bandiera et al., 2021)). To stratify randomization according to the risk of nonadherence due to the adverse effects of statins or the complexity of drug regimen, four randomization groups were created (i.e., patients monitored with at least a statin, patients monitored with at least one medication with multi-dose regimen, patients with both of the former conditions, and patients with none of the former conditions) (Bandiera et al., 2021).

#### 2.2.1 Intervention phase: *the* interprofessional medication adherence program (IMAP)

As part of the IMAP (Lelubre et al., 2015), all included patients used at least one electronic monitor (EM, Medication Event Monitoring System, MEMS, and MEMS AS, AARDEX Group, Sion, Switzerland), an interactive digital technology, to monitor their medication adherence. Each EM contained one oral prescribed chronic treatment. Monitoring priority was determined for each consecutive patient as follows: 1) antidiabetics, 2) antihypertensive drugs, diuretics, beta- and alpha-blockers, and calcium antagonists, 3) statins, and 4) aspirin. Before randomization, while investigators offered patients to monitor all eligible medications in EM, patients’ preferences on the number of EM to be used were taken into consideration so as not to burden their medication management habits. On the top of the EM cap, a liquid-crystal display (LCD) screen indicated the number of EM opening(s) during 24 h from 3:00 am to 2:59 am the day after. The EM registers the date and time of each EM opening, which is considered a proxy for the timing of drug intake. By reading EM data, the medAmigo™ software (AARDEX Group, Sion, Switzerland) establishes a chronology graph of medication intake during the current inter-visit intervention period. At each pharmacy visit, the pharmacist investigated EM deviation use by asking the patient to report i) non-monitored periods during which the medication was taken without opening the EM (i.e., during hospitalizations and holidays); ii) the use of pocket-doses (i.e., when the patient took a tablet outside of the EM to swallow it more than 24 h later) and curiosity checks (i.e., when the patient opened the EM without taking the dose); and iii) the usual time between EM openings and the medication intake. In addition, pharmacy technicians calculated an aggregated value of days covered by the pill count for conciliation with EM data. Any significant discrepancy was immediately investigated during the interview by the pharmacist.

The intervention consisted in face-to-face 15–20-min motivational interviews between the patient and the pharmacist based on the electronic adherence feedback presented in the form of a chronology plot. The intervention was built upon the Fisher et al. (2006) socio-cognitive theoretical framework “information–motivation–behavior”. Pharmacists and patients investigated together the patient’s habits and skills in self-managing medication and side effects. The pharmacist explored the patient’s own beliefs, preferences, and motivation to take the treatment and delivered information according to the patient’s needs. If necessary, goals for improving medication adherence were set collaboratively by the patient with the pharmacist, according to patient engagement, from one interview to the next interview. After the end of the interview, the pharmacist sent a report summarizing the content of the intervention to the healthcare team (i.e., endocrinologist, nephrologist, general practitioner, diabetes specialist nurses, psychologists, and dieticians).

#### 2.2.2 Post-intervention (follow-up) monitoring phase

After the end of the intervention phase, medication adherence was continuously monitored by EM until the end of the study (i.e., 24-month post-inclusion). During the post-intervention phase, the patient did not receive any intervention. EM data were blinded to the patient, the pharmacy team, the medical team, and the researchers. At each follow-up pharmacy visit, the pharmacist evaluated EM use deviations through the same set of questions as during the intervention phase and reported the answers in a case report form (CRF). In addition, pharmacy technicians counted pills left in the EM without calculating any adherence rate.

At each pharmacy visit during both the intervention and the follow-up phases, pharmacists measured prospectively patients’ systolic and diastolic blood pressures and heart rate, using a systematic methodology and a standardized device (e.g., measuring seated blood pressure on the same arm at each visit, after a 5-min rest period, measured three times, and then calculating a mean). They measured and reported patients’ abdominal circumference every 6 months. At 18 to 21 months post-inclusion in both groups, in patients still participating in the study, a blood sample allowed collecting laboratory values at this time point. In order to prevent patients from coming to the pharmacy during the lockdown enforced by the coronavirus disease (COVID)-19 pandemic (from March to June 2020), the medications were sent by mail so that patients could fill their EMs at home (Bourdin et al., 2022). The motivational interviews were delivered by phone, yet without EM feedback (Bourdin et al., 2022) for patients in the intervention phase. In order to guarantee homogeneity of the interventions between patients, the intervention phase was extended for 3 months after the lockdown in all patients (*n* = 15), who were in the intervention phase.

### 2.3 Database construction

#### 2.3.1 Collection of patients’ clinical and sociodemographic data

Patients’ demographic data (age, gender, marital status, ethnicity, and education level) and clinical data at inclusion (type of diabetes, time since diabetes diagnosis, body mass index (BMI), abdominal circumference, diagnosis of retinopathy, presence of atrial fibrillation, systolic and diastolic blood pressures and heart rate, eGFR decline per year, current or past diagnosis of depression or anxiety, smoking status, number of chronic treatments prescribed, and patient use of adherence support tools) were collected in patients’ electronic medical and administrative records.

The historical eGFR decline per year was calculated from patients’ blood creatinine concentrations available from 2000 to 2021, upon a previously described methodology (Bandiera et al., 2022a; Trucello et al., 2023).

The following clinical variables were collected for each patient as the mean of the values measured in the 12 months prior to study inclusion: glycated hemoglobin (HbA1c), eGFR, creatinine blood concentration, and low-density lipoprotein (LDL)-cholesterol. Missing data were clearly depicted. All data were collected on the secure web platform REDCap™ version 6.13.3 (Vanderbilt University) (Harris et al., 2009).

#### 2.3.2 EM adherence database

Patients’ EM raw data were cleaned and enriched using the CleanADHdata.R script (available on https://github.com/jpasquier/CleanADHdata), developed by our research team. The script truncates the EM database from the first to the last date of each EM use. The periods during which the EM was not used but the medication was taken (e.g., during holidays or hospitalizations) were set as non-monitored periods, and implementation was not calculated during these non-monitored periods. The number of pocket-doses reported by the patients was reconciled with pill count (i.e., the difference between the number of pills delivered and returned between two consecutive pharmacy visits) (Rotzinger et al., 2016). Covariables were inserted in each EM (e.g., the international nonproprietary name of the molecule monitored and its dose strength) and for each patient (e.g., randomization group, phase of the study, gender, and age).

### 2.4 Statistical analysis

#### 2.4.1 Descriptive analysis

Continuous sociodemographic and clinical variables were described by medians and interquartile ranges (IQRs) and qualitative data by proportions of patients in each group.

#### 2.4.2 Implementation and discontinuation

For each electronic monitor (EM) used by the patient, the medication intake is considered correct (= 1) a given day if the number of observed EM opening(s) is at least equal to the number of expected EM opening(s) based on the regimen provided in the prescription sheet and is considered incorrect otherwise (= 0). For every patient at each day of the monitoring period, an overall optimal medication intake (= 1) is defined by the product of each EM medication intake outcome: the medication intake needs to be correct (= 1) for all EM monitored in each drug class (i.e., antidiabetics, antihypertensive drugs, and statins) to consider a global correct medication intake for that day. Empirical medication implementation is then expressed as the proportion of patients with a global correct medication intake (proportion of outcomes = 1) at each day of the monitoring period among patients still participating in the study at that day.

From study inclusion to the end of the intervention (6 *versus* 12 months) and in the follow-up phase until 24 months post-inclusion, longitudinal implementation was described using the generalized estimating equation (GEE) model on the daily medication intake 0/1. Implementation was then estimated using the model for two representative patients: one who benefited from the intervention during 12 months (a patient from group A) *versus* one who benefited from the intervention during 6 months (a patient from group B). Implementation was estimated in three different GEE models, showing implementation to antidiabetics, antihypertensive drugs, and statins, respectively. The probability of treatment implementation was estimated for each drug class at 6, 12, 18, and 24 months for both representative patients A and B. The difference in implementation between both representative A and B patients (Δ) was presented with the 95% confidence interval (95% CI).

A discontinuation was defined when patients stopped taking at least one of their treatments earlier than planned by the prescriber, due to side effects or for any other patients’ unilateral and personal reasons. Other reasons for premature treatment stop (i.e., clinical reasons other than side effects) or study interruption without treatment discontinuation were considered censoring times. We represented graphically the moments of discontinuation in each model.

#### 2.4.3 Systolic and diastolic blood pressures

In patients treated with antihypertensive drugs, we analyzed systolic and diastolic blood pressures using linear mixed-effects models with polynomials of time.

The statistical analysis was performed using the statistical software R (R-development-core-team, 2005).

## 3 Results

### 3.1 Included patients

The PANDIA-IRIS study was offered to 275 patients, 73 of which accepted to participate. The main reasons for non-participation were investigated as part of the “participation to the PANDIA-IRIS” study, the results of which have been published elsewhere (Bandiera et al., 2022a). The sociodemographic and clinical variables of the 73 included patients at baseline (group A n = 35 and group B n = 38) are presented in Table 1. Most of the patients were male, Caucasian, diagnosed with type 2 diabetes, and had a basic- to intermediate-level schooling. Patients were polypharmacy, and most of them already used a weekly pillbox to manage a median of 9 chronic prescribed treatments. More than one-third of patients in each group were current smokers at study inclusion.

**TABLE 1:** Baseline demographic and clinical characteristics of patients included in the PANDIA-IRIS study.

	Group A (n = 35)	Group B (n = 38)
	12-month intervention	6-month intervention
Demographic data		
Age (years), median (IQR)	66.3 (58.9; 70.9)	62.0 (56.0; 69.2)
Female gender, n patients (%)	4 (11.4)	8 (21.1)
Marital civil status^a^, n patients (%)	16 (45.7)	16 (42.1)
Caucasian, n patients (%)	29 (82.9)	33 (86.8)
Education level, n patients (%)	Without training after mandatory school, 11 (31.4)	Without training after mandatory school, 6 (15.8)
	Professional training, 17 (48.6)	Professional training, 21 (55.3)
	General training, 3 (8.6)	General training, 1 (2.6)
	Higher education, 2 (5.7)	Higher education, 4 (10.5)
	Universities, 2 (5.7)	Universities, 6 (15.8)
Clinical data		
Type 2 diabetes^b^, n patients (%)	34 (97.1)	34 (89.5)
Time since diabetes diagnosis (years), median (IQR)	9.6 (4.7; 16.3)	9.1 (4.3; 18.8)
	Missing data n = 1	
BMI, median (IQR)	31.3 (27.6; 33.1)	31.9 (28.1; 34.7)
	Missing data n = 3	Missing data n = 3
Abdominal circumference (cm), median (IQR)	115 (105–122)	113 (100–119)
Diagnosis of retinopathy, n patients (%)	8 (22.9)	17 (44.7)
Presence of atrial fibrillation, n patients (%)	2 (5.7)	5 (13.2)
Systolic blood pressure (mmHg), median (IQR)	135 (125; 152)	133 (121; 143)
Diastolic blood pressure (mmHg), median (IQR)	71 (62; 80)	77 (69; 84)
Heart rate, median (IQR)	72 (62; 79)	78 (65; 86)
HbA1c (%), median (IQR)	7.6 (6.8; 8.2)	7.2 (6.8; 8.2)
	Missing data n = 4	Missing data n = 9
eGFR (mL/min/1.73 m^2^), median (IQR)	40 (34.2; 42.5)	43 (37.5; 52.6)
	Missing data n = 19	Missing data n = 18
eGFR decline per year (mL/min/1.73 m^2^/year), median (IQR)	−2.4 (−4.42; −0.29)	−2.4 (−4.27; −0.84)
Creatinine blood concentration (μmol/L), median (IQR)	128 (88.0; 154.5)	120 (97.5; 147.1)
LDL-cholesterol (mmol/L), median (IQR)	2.2 (1.85; 2.65)	2.2 (1.50; 2.60)
	Missing data n = 7	Missing data n = 9
Current or past diagnosis of depression or anxiety, n patients (%)	11 (31.4)	6 (15.8)
Current smokers, n patients (%)	11 (31.4)	14 (36.8)
Number of prescribed chronic medications, median (IQR)	9 (7–12)	9 (7–12)
Previous use of adherence tools, n patients (%)	21 (60.0)	19 (50.0)
Adherence personal tools used among those who had used an adherence tool, n patients (%)	Electronic pillbox, 6(28.6)	Electronic pillbox, 5 (26.3)
	Weekly pillbox, 19 (90.5)	Weekly pillbox, 14 (73.7)
	Personal items, 2 (9.5)	Personal items, 3 (15.8)
	No adherence tools used, n = 14	No adherence tools used, n = 19
Stratification list, n patients (%)	Statins, n = 10	Statins, n = 11
	Multi-dose regimen, n = 8	Multi-dose regimen, n = 10
	Statins and multi-dose regimen, n = 12	Statins and multi-dose regimen, n = 12
	No statin nor multi-dose regimen, n = 5	No statin nor multi-dose regimen, n = 5
Number of EMs dispensed, n patients (%)	1 EM, n = 5 (14.3)	1 EM, n = 4 (10.5)
	2 EMs, n = 9 (25.7)	2 EMs, n = 15 (39.5)
	3 EMs, n = 11 (31.4)	3 EMs, n = 5 (13.2.)
	4 EMs, n = 8 (22.9)	4 EMs, n = 10 (26.3)
	5 EMs, n = 1 (2.9)	5 EMs, n = 3 (7.9)
	6 EMs, n = 1 (2.9)	6 EMs, n = 1 (2.6)
Number of EMs used per patient, median (IQR)	3 (2–4)	2.5 (2–4)

NB: EM, electronic monitor; IQR, interquartile range; BMI, body mass index; LDL, low-density lipoprotein; HbA1c, glycated hemoglobin; eGFR, estimated glomerular filtration rate.

^a^ The other patients are in partnership, separated, divorced, widow, or single.

^b^ The other patients have diabetes type 1, latent autoimmune diabetes in adults, post-transplantation, or glucocorticoid-induced diabetes. From October 2019, the eligibility criteria were expanded to include types of diabetes other than type 2, which explains the low proportion of patients in these categories.

For patients still in the study at 18–21 months, from whom a blood sample was collected, the median glycated hemoglobin (HbA1c) was 7.6% (IQR 7.1; 7.9) in group A (n = 15 patients) and 7.8% (6.6; 8.4) in group B (n = 12 patients). The median albumin–creatinine ratio (ACR) was 15.6 mg/mmol (IQR: 4.7; 38.7) in group A (n = 14 patients) and 5.8 mg/mmol (IQR: 2.8; 38.0) in group B (n = 6 patients). The median LDL-cholesterol level was 1.6 mmol/L (IQR: 1.5; 2.4) in group A (10 patients) and 1.8 mmol/L (IQR: 1.3; 2.4) in group B (n = 7 patients).

At study inclusion, the eGFR decline (−2.4 mL/min/1.73 m^2^/year in both groups) was faster in patients included in our study compared to patients with type 2 diabetes in the Swiss ambulatory care (−1.2 mL/min/1.73 m^2^/year (standard deviation (SD) 0.05) in men and −1.0 mL/min/1.73 m^2^/year (SD 0.06) in women (Lamine et al., 2016)).

**FIGURE 2:**
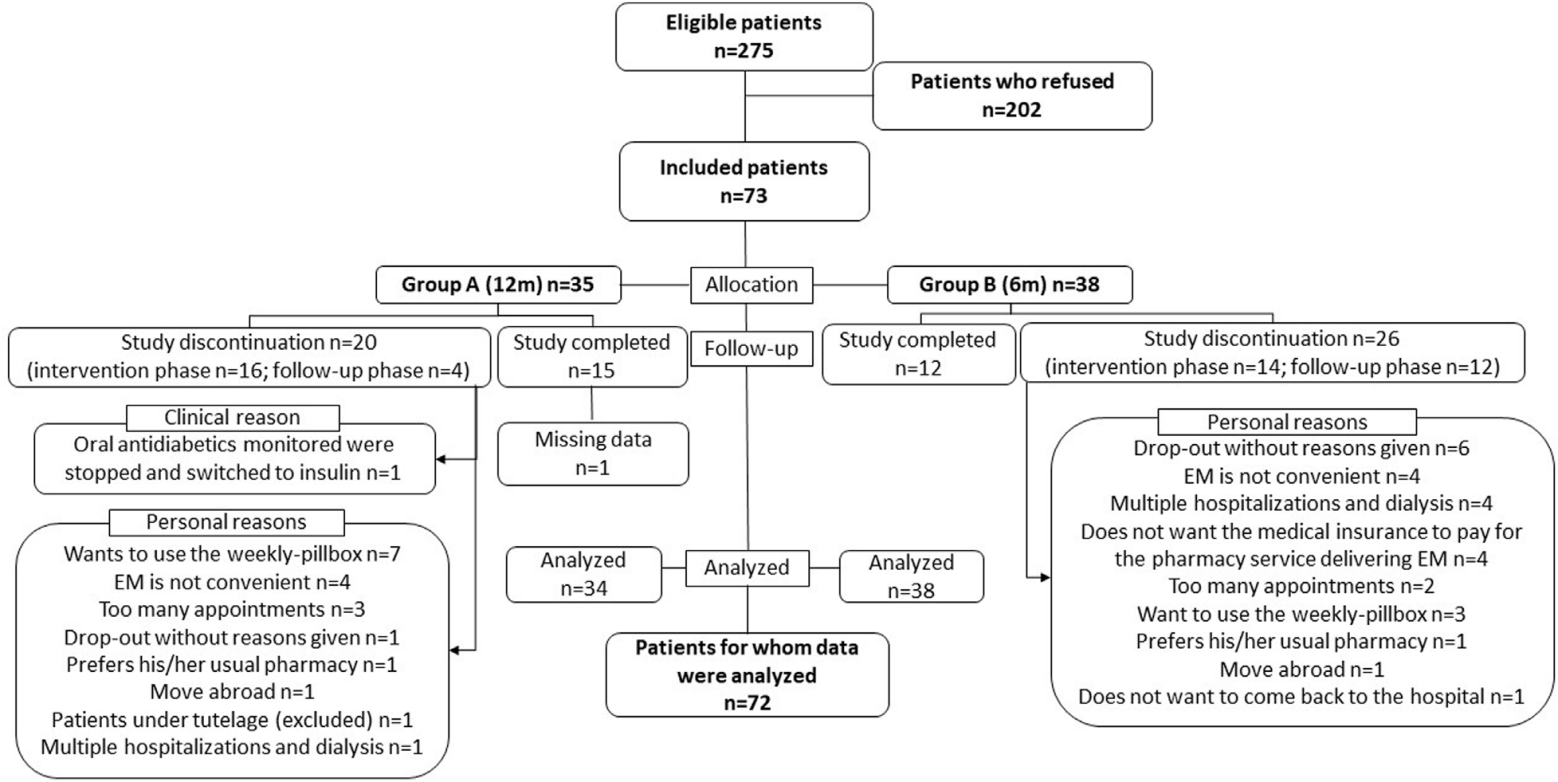
Flow of patients from enrollment to data analysis in the PANDIA-IRIS study. EM, electronic monitor.

Figure 2 shows patient enrollment and follow-up in the study. In groups A and B, respectively, 20 and 26 patients dropped out, mainly due to logistical reasons or because the study was perceived as an additional burden in their care. Of note, patients’ satisfaction about the intervention was reported elsewhere (Bandiera et al., 2022a). Patients in groups A and B spent, respectively, a median time of 539 days (IQR 124; 747) and 366 days (IQR 145; 740) in the study. The EM data of one patient included in group A were not analyzed as the patient used a weekly pillbox instead of the EM. After completion of the study at 24 months, 4 *versus* 3 patients in groups A and B, respectively, decided to continue attending the routine IMAP. There was no patient with an electronic medication implementation of less than 30% for at least one medication throughout two successive pharmacy visits during the post-intervention phase.

### 3.2 Medication implementation by drug classes

#### 3.2.1 Implementation to antidiabetics and antihypertensive drugs

**FIGURE 3:**
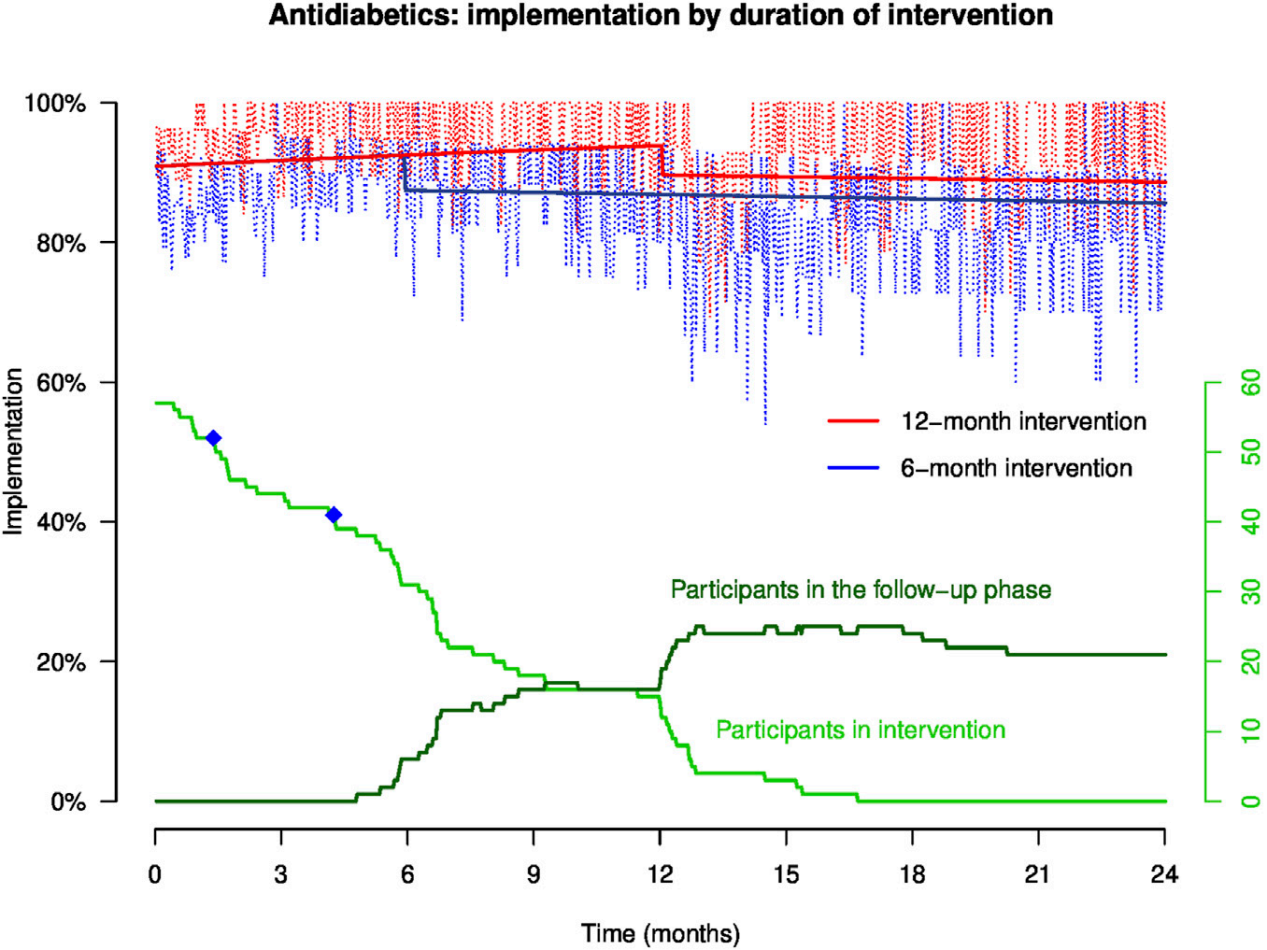
Implementation to antidiabetics in representative patients of groups **(A, B)** throughout the study. NB: the light red and blue curves show empirical implementation, and the thick red and blue lines represent implementation to antidiabetics modeled by GEE. The green curves represent the number of participants over time in the intervention and the follow-up phases, and the blue dots on the green curve show the moment when patients discontinued at least one of their antidiabetics.

**FIGURE 4:**
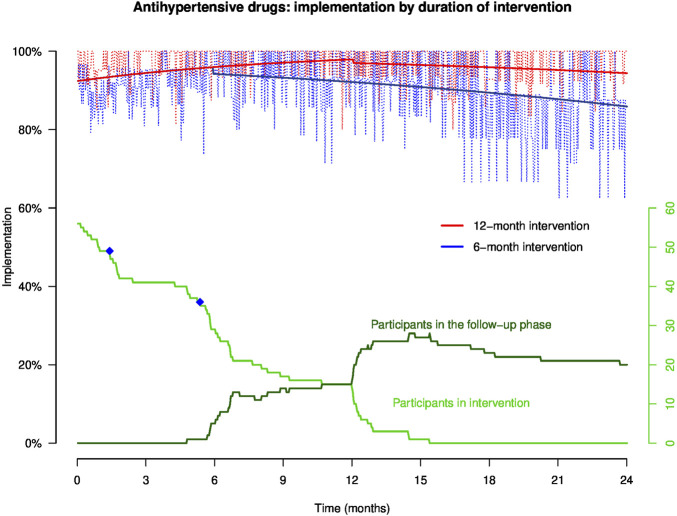
Implementation to antihypertensive drugs in representative patients of groups **(A, B)** throughout the study. NB: the light red and blue curves show empirical implementation, and the thick red and blue lines represent implementation to antihypertensive drugs modeled by GEE. The green curves represent the number of participants over time in the intervention and the follow-up phases, and the blue dots on the green curve show the moment when patients discontinued at least one of their antihypertensive drugs.

Empirical implementation to antidiabetics and antihypertensive drugs in patients who were prescribed antidiabetics (n = 57, 28 patients in group A and 29 patients in group B) and antihypertensive drugs (n = 57, 25 patients in group A and 32 patients in group B) is presented in Figures 3 and 4, respectively. Not enough patients were treated by aspirin (n = 6, 2 patients in group A and 4 patients in group B) to allow a reliable analysis of implementation to aspirin. The equations of the GEE models are presented in Supplementary Material S1.

The GEE models represent implementation for a representative patient participating in the intervention during 12 months (red line) or 6 months (blue line). Patient implementation to antidiabetics and antihypertensive drugs increases steadily during the intervention period. At the end of the intervention (at 6- and 12-month post-inclusion), the model shows that implementation drops and then gradually decreases over time during the follow-up phase. In this follow-up phase, patients who benefited from the intervention during 12 months maintained a higher implementation than patients who received the intervention during 6 months. At 6, 12, 18, and 24 months, implementation to antidiabetics and antihypertensive drugs in patients who benefited from the intervention for 12 months was continuously higher than that in patients who received the intervention for 6 months.

At 12 months, implementation to antidiabetics in a patient completing the 12-month intervention (patient A) was statistically higher than that in a patient who completed the 6-month intervention 6 months earlier (patient B) (93.8% *versus* 86.8%; Δ 7.0%, 95% CI: 5.7%; 8.3%) (Table 2). At 12 months, implementation to antihypertensive drugs in patient A was also higher *versus* patient B (97.9% *versus* 92.1%; Δ 5.8%, 95% CI: 4.8%; 6.7%).

**TABLE 2:** Implementation to antidiabetics, antihypertensive drugs, and statins at 6, 12, 18, and 24 months post-inclusion.

	Time since inclusion, months (m)	Number of patients	Implementation in group A (intervention lasted 12 months) (%)	Implementation in group B (intervention lasted 6 months) (%)	Difference (Δ) (%)	95% CI (%)	
Antidiabetics	6	37	92.5	87.4	5.1	3.7	6.5%
	12	31	93.8	86.8	7.0	5.7	8.3%
	18	24	89.1	86.2	2.9	1.7	4.1%
	24	21	88.6	85.6	3.0	1.7	4.4%
Antihypertensive drugs	6	34	95.9	94.2	1.7	0.7	2.8%
	12	30	97.9	92.1	5.8	4.8	6.7%
	18	23	95.9	89.5	6.5	5.4	7.6%
	24	20	94.4	85.9	8.5	6.6	10.7%
Statins	6	31	95.4	96.4	−1.1	−1.8	−0.2%
	12	25	95.0	95.4	−0.4	−1.7	0.7%
	18	18	95.1	94.2	0.9	0.1	1.8%
	24	14	93.7	92.6	1.1	0.1	2.4%

NB: CI, confidence interval.

At 24 months, implementation to antidiabetics in patient A was statistically higher compared to that in patient B (88.6% *versus* 85.6%; Δ 3.0%, 95% CI: 1.7%; 4.4%), and implementation to antihypertensive drugs was also higher in patient A than B (94.4% *versus* 85.9%; Δ 8.5%, 95% CI: 6.6%; 10.7%) (Table 2).

No patient of group A *versus* three patients of group B discontinued at least one of their monitored treatments. The moments of treatment discontinuation are shown in Figures 3 and 4 by the blue dots on the green curve showing the number of participants over time.

#### 3.2.2 Implementation to statins

**FIGURE 5:**
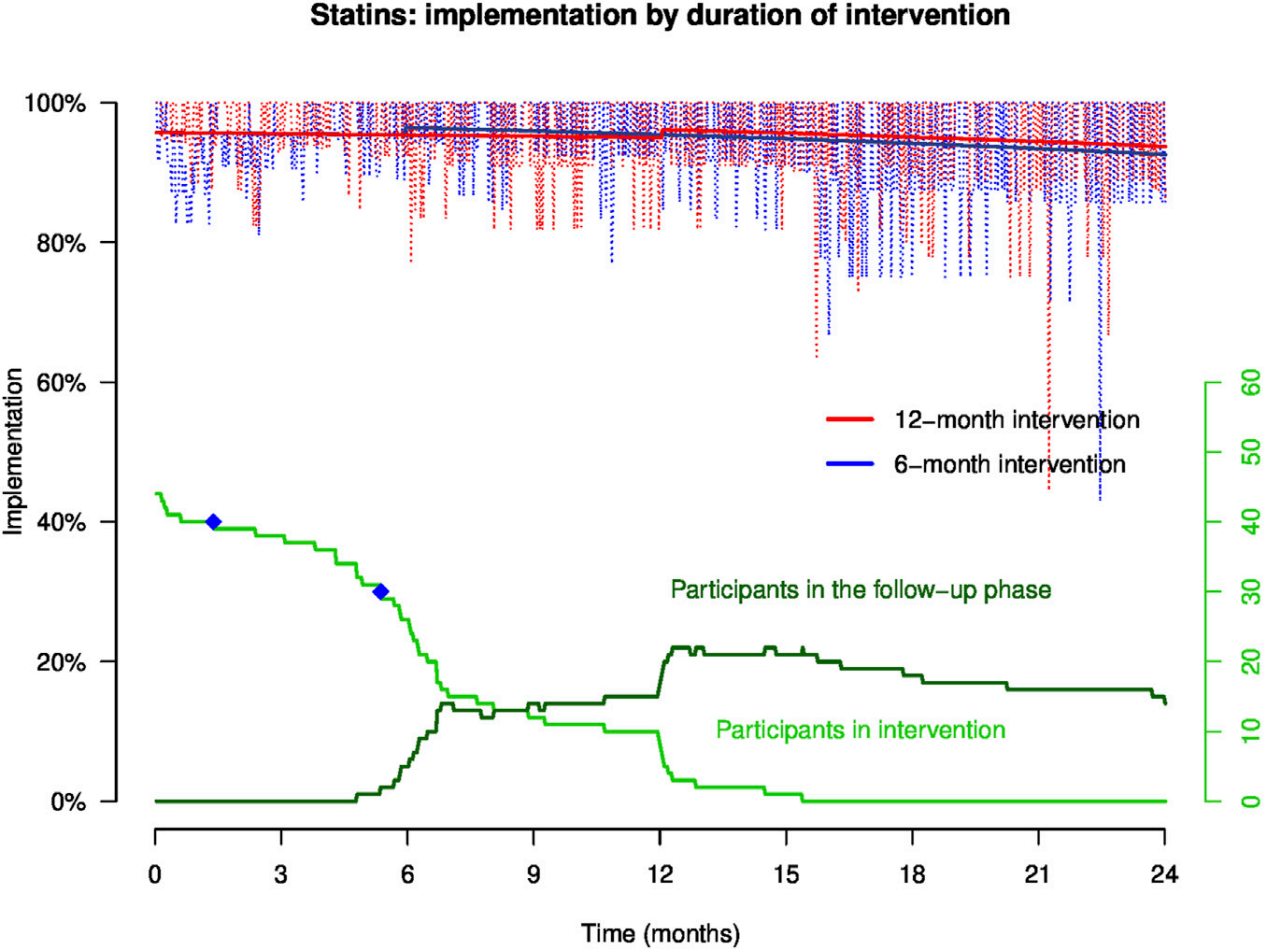
Implementation to statins in representative patients of groups **(A, B)** throughout the study. NB: the light red and blue curves show empirical implementation, and the thick red and blue lines represent implementation to statins modeled by GEE. The green curves represent the number of participants over time in the intervention and the follow-up phases, and the blue dots on the green curve show the moment when a patient discontinued the statin.

Empirical implementation in patients who were prescribed statins (n = 44, 20 patients in group A and 24 patients in group B) and implementation to statins modeled by GEE are presented in Figure 5. Implementation remained stable during the intervention until 12 months. At the end of the intervention at 6 and 12 months post-inclusion, implementation increases slightly and then decreases steadily in the follow-up phase. At 12 months, implementation to statins in a representative patient of group A *versus* B was, respectively, 95.0% and 95.4% (Δ −0.4%, 95% CI: −1.7%; 0.7%). At 24 months, implementation to statins was comparable between both representative patients: implementation was 93.7% in patient A and 92.6% in patient B (Δ 1.1%, 95% CI: 0.1%; 2.4%).

### 3.3 Office systolic and diastolic blood pressure

During the coronavirus disease (COVID)-19 pandemic lockdown in 2020, we had to stop collecting blood samples from patients for research purposes. Therefore, numerous laboratory data were missing at different time points (e.g., HbA1c, eGFR, LDL-cholesterol, and ACR), which prevented us from analyzing the impact of medication adherence on clinical outcomes and from calculating the UKPDS and the ADVANCE clinical scores.

**FIGURE 6:**
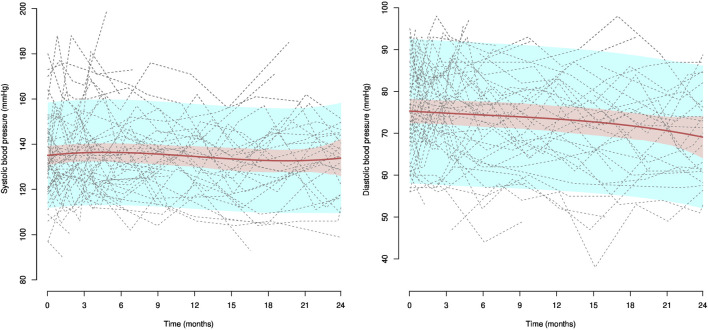
Systolic and diastolic blood pressure in patients treated with antihypertensive drugs. NB: dotted lines represent individual trajectories, and confidence intervals around the predicted mean are presented in pink and the prediction intervals in blue.

The estimated tendency of individual systolic and diastolic blood pressures for the 57 patients treated with antihypertensive drugs (25 patients in group A and 32 patients in group B) is presented in Figure 6, along with confidence and prediction intervals and all individual blood pressure trajectories. A slight downward trend was observed for systolic and diastolic blood pressures. At inclusion, systolic blood pressure was estimated at 135.1 mmHg (95% CI: 130.7; 139.6), while the estimation was 136.3 mmHg (95% CI: 131.6; 140.8) at 6 months, 134.5 mmHg (95% CI: 129.8; 139.3) at 12 months, 132.8 mmHg (95% CI: 127.9; 137.7) at 18 months, and 133.9 mmHg (95% CI: 125.5; 142.3) at 24 months. At inclusion, diastolic blood pressure was estimated at 75.3 mmHg (95% CI: 72.3; 78.2), while the estimation was 74.4 mmHg (95% CI: 71.6; 77.3) at 6 months, 73.4 mmHg (95% CI: 71.6; 77.3) at 12 months, 71.8 mmHg (95% CI: 68.7; 75.0) at 18 months, and 69.0 mmHg (95% CI: 64.5; 74.0) at 24 months. No differences were observed between groups (data not shown).

## 4 Discussion

### 4.1 Main results

The pharmacist-led interprofessional medication adherence program improved implementation to antidiabetics and antihypertensive drugs but not to statins in patients with DKD, and the effect persisted 24 months after inclusion. Therefore, the duration of the intervention is important to ensure a lasting effect on the maintenance of medication implementation: the longer the patients benefit from the intervention, the more the implementation increases over time, and the more the effect lasts after the end of the intervention. Office blood pressure decreased slightly over time, but no difference was observed between groups.

### 4.2 Effect of the IMAP on implementation

The effect of the IMAP on implementation to antihypertensives and antidiabetics was significant, whereas no change was observed in the implementation to statins. This can be explained by several hypotheses. First, antidiabetics and antihypertensive drugs are often prescribed with a regimen more complex than that of statins (i.e., multiple drug intakes per day), whereas statins are mostly prescribed with a once-daily regimen. In addition, drug, dose, and regimen changes occurred more often with antidiabetics and antihypertensive drugs than with statins. These factors may contribute to the difficulty for patients to adhere optimally to antidiabetics and antihypertensive drugs compared to statins, and the room for improvement in treatment implementation may be larger in these drug classes than with statins. Patients were used to taking their statins for several years, and there was no major complaint on usual statin side effects. Second, as the implementation to statins was already high (>95%) at study start in both groups, pharmacists focused the discussion more on antidiabetics and antihypertensive drugs during the motivational interviews than on statins (based on qualitative study monitoring data).

### 4.3 Definition of treatment implementation

Our definition of treatment implementation states that patients need to implement optimally all their medications monitored on day x in order to have an overall optimal medication implementation at day x. This definition has been commonly used in previous research studies (Schneider et al., 2019; Bandiera et al., 2022b; Pasquier et al., 2022). However, patients with DKD are polypharmacy, and patients included in the PANDIA-IRIS study often used more than one EM. Each additional EM used reduced the probability to have a daily optimal overall implementation. For instance, in a patient who used five EMs and optimally implemented four of these, the overall implementation would depend on the implementation of the fifth treatment, leading to an underestimation of the actual treatment implementation. As the number of EMs used per patient is distributed evenly in our sample, probably reinforced by the stratification of the randomization, our analysis is valid. Additionally, we analyzed medication implementation by drug classes to limit the risk. Nevertheless, the definition of treatment implementation monitored through EM with the binary variable 1/0 needs to be further adapted for polypharmacy patients. The probability of an optimal implementation could be determined by the ratio of treatments taken optimally to the total number of monitored treatments (i.e., on day x, if patients have an optimal implementation to 4/5 of their medications, the probability of an optimal implementation at day x would be 80%). Our research raises the point that the operational definitions of implementation should be evaluated further in polypharmacy patients, as well as the statistical methodology (Pasquier et al., 2022).

### 4.4 Effect of the study on clinical practice

Patients were overall satisfied about the IMAP (Bandiera et al., 2022a). A substantial number of patients left the study during the intervention phase, mainly owing to personal reasons (cf. Figure 2, i.e., 46% (16/35) of patients in the 12-month intervention *versus* 37% (14/38) of patients in the 6-month intervention). This important number of dropouts shows the difficulty in conducting behavioral interventions in routine practice. To improve retention in the intervention while limiting the inclusion bias, interventions such as the IMAP should be considered an integrated component of the standard of care for polypharmacy patients. Including all consecutive chronically ill patients to the IMAP would allow a prospective evaluation of the effect of the IMAP on clinical outcomes. For example, early in their therapeutic itinerary, polypharmacy patients would be invited to experience the IMAP for 12 months to co-construct their medication adherence with healthcare providers, tailored to their individual needs, before deciding whether they would benefit from continuing the intervention or repeating it later based on defined clinical outcomes, personal experiences, and indicators (Bandiera et al., 2022a). The interprofessional collaborations between patients, pharmacists, physicians, nurses, and other healthcare providers should be strengthened in order to synergistically promote the IMAP to patients and to better define the roles and responsibilities of each healthcare provider in supporting medication adherence (Bandiera et al., 2022c).

A trend toward a decrease in blood pressure was observed, which may be related to improved adherence to treatment. However, differences in blood pressure between groups were not significant. The sample size was probably too small to draw any firm conclusion on the effect of a difference of 3%–5% in implementation to antihypertensive drugs on blood pressure. Accurate modeling of blood pressure as a function of adherence levels is needed as a decision aid for patients and healthcare professionals to better characterize the expected clinical benefit in relation to patient’s adherence effort (Polychronopoulou et al., 2021).

### 4.5 Limitations and strengths of the study

The strengths of the PANDIA-IRIS study are described as follows: first, the IMAP is a proven, theory-based, semi-structured intervention program implemented in routine practice. As part of the intervention, pharmacists i) explore patients’ capability to acquire knowledge and skills to strengthen their self-efficacy, ii) explore and participate in the development of patients’ motivation to take the treatment, and iii) explore opportunities in the patients’ environment that encourage behavioral changes to improve or maintain medication adherence. These three components affect patient health behaviors and are the main components of the Behavior Change Wheel model designed by Michie et al. (2011) to lead effective interventions.

Second, the design of the PANDIA-IRIS study is innovative as it provides an analysis of the duration of an intervention, which is insufficiently studied in the literature, by comparing between 6- and 12-month interventions. To the best of our knowledge, this is also the first time that medication adherence was monitored during an extended period of time of more than a year (24 months), including the post-intervention period in order to understand the durability of the intervention. Our results suggest that medication adherence interventions should be delivered over long periods of time based on patients’ needs by adapting the level of the intervention to short-, middle-, and long-term objectives. Our experience with the IMAP in routine care shows that some chronic patients stay in the program for years, whereas others leave it after a semester and sometimes return afterward.

Third, we reported findings through a robust methodology. We used electronic monitoring for 24 months, which is considered the most robust methodology to objectively and longitudinally measure medication implementation over time providing an adherence history. The statistical analysis procedures used on repeated adherence electronic monitoring measures were previously developed and validated (Schneider et al., 2019; Pasquier et al., 2022), and the analysis of the implementation to the different drug classes allowed determining the differential effect of the IMAP on implementation to antidiabetics, antihypertensive drugs, and statins.

Some limitations are to be acknowledged. First, even if the target sample size was reached, a significant number of patients either refused inclusion or dropped out during the study. Refusal to enroll has been addressed previously (Bandiera et al., 2022a). Our high level of patient adherence since inclusion may indicate a possible selection bias. This bias is difficult to address in clinical practice, unless a medication adherence program is embedded in usual clinical practice because “the very people with the worst adherence may be the least likely to accept inclusion in a non-routine medication adherence program” to paraphrase the famous quote by Tourangeau and Smith (1996): “The very persons with the most sensitive information to report may be the least likely to report it” (Tourangeau and Smith, 1996).

Regarding dropouts, we cannot exclude that patients who refused to participate or who left the study had a different medication adherence than those who completed the study. This needs further exploration.

Second, patients used the EM during the follow-up period, which could have been a supportive tool in their medication management. The LCD screen on the top of the EM cap indicated the number of daily EM opening(s), which can help prevent forgetfulness. Furthermore, patients had to refill their EMs at the pharmacy every 3 months, and they were recalled by phone calls if they missed the appointment, which is not the standard of care. Pharmacists had to check EM use deviation at each follow-up visit for methodological reasons, which may have raised patient awareness on medication adherence during the follow-up period. In addition, the repeated blood pressure and the abdominal circumference measured by pharmacists during the follow-up phase may have influenced patient medication adherence. Thus, medication adherence measured during the follow-up period might have been higher than that in the standard of care.

Third, owing to the low prevalence of treatment discontinuations, we did not analyze medication persistence. We would need a larger database with a larger sample size to carefully evaluate the effect of the IMAP on medication persistence in patients with DKD after the intervention. Finally, the number of blood pressure measurements collected was limited to the number of pharmacy visits. The individual variability in blood pressure over time was high; the ambulatory blood pressure measurements would have provided a more accurate evaluation of blood pressure control over 24 h than the office blood pressure. Future studies should increase the number of data collected and organize a retrospective collection of blood pressure measurements during the 12 months before the intervention to better describe blood pressure trajectories.

## 5 Conclusion

The interprofessional medication adherence program (IMAP) supports adherence in terms of implementation to antidiabetics and antihypertensive drugs in patients with diabetic kidney disease. The longer the patients benefit from the intervention, the more the implementation increases over time, and the more the effect lasts after the end of the intervention. The IMAP should be recommended for at least 12 months, or longer, with the intensity adjusted depending on the needs of the patients, to have a positive and sustained effect on treatment implementation in patients with diabetic kidney disease. The effect on clinical outcomes needs to be further investigated in the long term.

## Data Availability

The datasets for this article are not publicly available due to concerns regarding participant/patient anonymity. Requests to access the datasets should be directed to the corresponding author.

## Abbreviations

BMI, body mass index; CHUV, Centre hospitalier universitaire vaudois; CI, confidence interval; CONSORT, Consolidated Standards of Reporting Trials; COVID, coronavirus disease; CRF, case report form; DKD, diabetic kidney disease; eGFR, estimated glomerular filtration rate; EMs, electronic monitors; EMERGE, ESPACOMP Medication Adherence Reporting Guidelines; GEE, generalized estimating equation; HbA1c, glycated hemoglobin; IMAP, interprofessional medication adherence program; IQR, interquartile range; LADA, latent autoimmune diabetes in adults; LCD, liquid-crystal display; LDL, low-density lipoprotein; PANDIA-IRIS, Patients diabétiques et insuffisants rénaux: un programme interdisciplinaire de soutien à l’adhésion thérapeutique; PART-PANDIA, participation to the PANDIA-IRIS study; SD, standard deviation.
