# Supplementary material for: The differential impact of a 6-versus 12-month pharmacist-led interprofessional medication adherence program on medication adherence in patients with diabetic kidney disease: the randomized PANDIA-IRIS study
Source: Front Pharmacol. 2024 Jan 24;15:1294436. doi: 10.3389/fphar.2024.1294436 (PMC10847300; doi:10.3389/fphar.2024.1294436)
Supplement: Supplementary file 1 [file DataSheet1.pdf]

Supplementary Material 1: GEE formula equations

GEE analysis with antidiabetics:

$$Logit\big(\Pr(Z_j)\big) = 2.3 + 0.001 \cdot T1_j - 0.569 \cdot Jump_j - 0.0003 \cdot T0_j$$

GEE analysis with antihypertensive drugs:

$$Logit\big(\Pr(Z_j)\big) = 2.5 + 0.004 \cdot T1_j - 0.367 \cdot Jump_j - 0.002 \cdot T0_j$$

GEE analysis with statins:

$$Logit\big(\Pr(Z_j)\big) = 3.1 + 0.0004 \cdot T1_j - 0.271 \cdot Jump_j - 0.001 \cdot T0_j$$

Z_j = medication intake at day *j* (1=correct / 0=incorrect)

$T1_j$ = Time already spent in intervention at day *j* (in days)

$T0_j$ = Time already spent in follow-up at day *j* (in days)

$Jump_j$ = change of phase (from intervention to follow-up) at day *j* (1=change / 0=no change)
